# Gait Spatio-Temporal Parameters Vary Significantly Between Indoor, Outdoor and Different Surfaces

**DOI:** 10.3390/s25051314

**Published:** 2025-02-21

**Authors:** Lorenzo Brognara, Alberto Arceri, Marco Zironi, Francesco Traina, Cesare Faldini, Antonio Mazzotti

**Affiliations:** 1 Department of Biomedical and Neuromotor Sciences (DIBINEM), University of Bologna, 40127 Bologna, Italy; lorenzo.brognara2@unibo.it (L.B.); alberto.arceri@ior.it (A.A.); marco.zironi2@studio.unibo.it (M.Z.); francesco.traina@ior.it (F.T.); cesare.faldini@ior.it (C.F.); 2 1st Orthopaedics and Traumatologic Clinic, IRCCS Istituto Ortopedico Rizzoli, 40136 Bologna, Italy; 3 Chirurgia Protesica e dei Reimpianti di Anca e Ginocchio at the IRCCS Istituto Ortopedico Rizzoli, 40136 Bologna, Italy

**Keywords:** wearable sensor, gait, outdoor, gait speed, fall, stride length

## Abstract

Human gait is usually studied in clinical environments, but wearable devices have extended gait analysis beyond traditional assessments. Older adults tend to walk differently indoors and outdoors; however, most gait assessments are conducted on indoor surfaces. It is therefore important to evaluate gait in various outdoor environments. Insights gained from these assessments significantly enhance our understanding of the impact of environmental factors on gait performance and ensure that clinical evaluations are effectively aligned with everyday locomotion. A total of 100 participants with foot pain, 38 young (18–45 years) and 62 older adults (65–80 years), completed a 10-Metre Walk Test (10MWT) in three randomised conditions at their typical, comfortable walking pace, including (1) 10MWT of indoor walking, (2) 10MWT of outdoor walking on grass and (3) 10MWT of outdoor walking on a sidewalk. Wearable inertial sensors recorded gait data and the magnitudes of the following gait measures: gait speed, cadence, stride length, stride duration and asymmetry. A statistical analysis using ANOVA and post hoc comparisons revealed a significantly lower gait speed (*p* < 0.001), lower stride length (*p* < 0.001) and lower asymmetry (*p* < 0.001) indoors compared to outdoors, demonstrating that environmental factors significantly affect spatio-temporal gait parameters. Wearable sensor-based gait analysis performed in controlled clinical settings may underestimate real-life conditions. Some important spatio-temporal parameters, useful in detecting people with gait impairment and at risk of falling, are significantly affected by environment and individual postural ability more than demographic factors.

## 1. Introduction

Walking in the real world involves the integration of multiple tasks and requires cognitive flexibility to respond to environmental stimuli [1,2]. Gait disorders are increasingly associated with adverse events such as physical disability, falls, institutionalisation, and mortality [3,4].

Gait analysis is typically conducted in laboratories using motion capture and force plates, but time and financial constraints limit their use in clinical practice [5]. Moreover, testing on a flat, clean surface does not accurately reflect real-life conditions [6,7]. Daily activities involve challenging environments, leading to changes in gait patterns on different surfaces [8,9]. For these reasons, clinicians are focusing on collecting gait data in real-world environments, and wearable devices will enhance our understanding of daily gait patterns on different surfaces [10,11]. Objective evaluation in real-life conditions could help identify the causes of gait problems in people with foot and ankle disorders and guide the development of appropriate physical treatments based on recorded clinical outcomes [12].

A quantitative gait analysis is a crucial clinical tool for diagnosing gait abnormalities and evaluating treatment effectiveness [12,13,14]. Wearable devices provide a valuable opportunity to measure movement patterns in real-world environments [15]. To ensure valid and reliable data, it is important to investigate how spatio-temporal parameters change across different surfaces. Recent technological advancements have led to affordable, wearable sensors for gait analysis in everyday settings [16]. These sensors diagnose walking impairments in individuals with gait disabilities and are crucial for immediate control and rehabilitation therapies.

The aim of our study is to evaluate changes in the biomechanical characteristics of gait by measuring different spatio-temporal gait parameters in order to compare walking on three different surfaces and environments, namely a flat and clean surface indoors, a sidewalk outdoors, and an outdoor grass surface, in individuals with foot pain. Foot pain is one of the most prevalent symptoms associated with lower limb issues, especially among elderly individuals [17]. Foot pain can hinder mobility and balance, impacting walking in various environments, reducing physical functioning, lowering quality of life and increasing the risk of falls [18,19,20,21,22]. Additionally, we aimed to analyse whether the impact of the environment differed according to demographic factors, such as age and gender, or clinical characteristics, such as history of falls and postural stability.

## 2. Materials and Methods

### 2.1. Study Design and Participant Selection

A cross-sectional study was carried out between 10 January and 31 August 2024 on individuals attending the Academic Orthopaedic Clinic of the University of Bologna IRCCS Rizzoli Orthopaedic Institute in Bologna, Italy. All participants underwent a gait analysis on different surfaces, with a number of parameters being measured by sensors.

Approved by the Ethics Committee of University of Bologna (reference: 659/2021/Sper/IOR), participants were recruited through a research proposal. Each participant was informed by telephone about the aims of the study and gave informed consent before participation.

The inclusion criteria were (1) individuals of both genders; (2) individuals aged 18 or older; (3) individuals with foot pain. The exclusion criteria were (1) severe cognitive impairment or uncontrolled psychiatric issues; (2) active foot ulcers; (3) cancer; (4) retinopathy; (5) Charcot foot; (6) lower limb injuries or fracture in the past six months; (7) orthopaedic lower limb surgery in the past year.

Data were also collected on the subjects’ age, gender and history of falls. The Tinetti Scale was also used to assess the participants’ risk of falling. This scale is a useful tool for classifying functional ability, focusing on balance and gait. It consists of a gait section with seven items (maximum score of 12 points) and a balance section with nine items (maximum score of 16 points), making a total of 28 points. Scores are categorised as follows: 25–28 points indicate a low risk of falls; 19–24 points mean a moderate risk of falls and less than 19 points mean a high risk of falls [23,24].

### 2.2. Gait Assessment

Gait assessment was performed using a 10-Metre Walk Test (10MWT) while wearing portable inertial sensors. The 10MWT is a widely validated monitoring tool used to detect gait impairment and represent an alternative to the 6-Min Walk Test for people with relatively good walking ability [25,26,27,28]. Furthermore, the use of the 6MWT can be strenuous for older individuals or those with foot pain, and it impacts time efficiency [28].

Participants walked at their preferred speed while their gait parameters were assessed using Wiva Sciences (Wiva Science-LetSense Srl, Bologna, Italy), a widely used system that incorporates inertial measurement units (IMUs) worn on the lower back and secured with a sacroiliac belt [29,30,31,32,33,34,35,36,37,38]. Equipped with a tri-axial accelerometer, gyroscope and magnetometer, the sensors provide valuable quantitative data on individual performance, enhancing clinical interpretations and treatment evaluations. This system, manufactured by Letsense^®^ (Letsense Group, Bologna, Italy), provides spatial-temporal parameters and accurately estimates kinematic data, including position, acceleration and speed. It has been previously validated and compared in the gait analysis laboratory of the Rizzoli Orthopaedic Institute [39]. The device employs a micro-electro-mechanical system (MEMS) to detect movement and convert it into an electrical signal. The Biomech Studio software (Version 1.6.1.14687, LetSense Group srl., Bologna, Italy) automatically generates a gait analysis report based on the sensor input, presenting spatio-temporal and specific kinematic parameters, including:

- Cadence [steps/min]: The number of steps per minute.
- Stride length [m]: Distance between the heel contacts of the same foot.
- Gait speed [m/min]: Ratio between the length and duration of the stride.
- Stride duration [s]: The interval between sequential initial heel contacts by the same limb; time taken to complete one full gait cycle, from the initial contact of one foot to its subsequent contact.
- Asymmetry [%]: Stride length difference between right and left limbs during walking.
- Swing phase [%]: The swing phase occurs when the reference foot is not on the ground, accounting for about 40% of the gait cycle.
- Single-support duration [%]: The percentage of the gait cycle during which only one foot is in contact with the ground, typically representing about 40% of the cycle.
- Double-support duration [%]: The percentage of the gait cycle during which both feet are in contact with the ground, approximately 20% of the stance phase.

**Figure 1:**
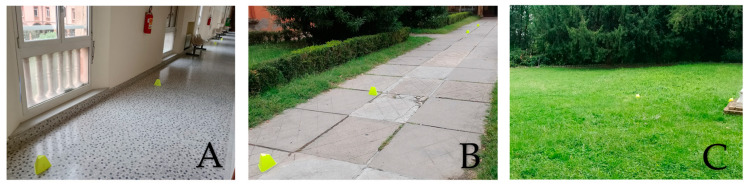
The three experimental environments: indoor on a flat and clean surface (**A**), outdoor on concrete sidewalk (**B**) and outdoor on grass (**C**).

Participants in the study were instructed to walk on three distinct surfaces as part of the assessment process. Participants explored three paths within the institute. To prevent bias and reduce learning effects, the testing sequence was randomised and clinicians were unaware of previous performance. Each path included guidance to the acceleration and deceleration zones, marked three metres before and two metres after the cones. These surfaces included a flat and clean indoor area (Figure 1A), which provided a controlled environment, an outdoor concrete pavement that simulated a typical urban environment (Figure 1B) and an outdoor grass area that represented more natural terrain (Figure 1C). Each walking session on these surfaces was performed in a randomised order to ensure the reliability of the results.

### 2.3. Statistical Analysis

Data collection was carried out utilising Microsoft Excel (Microsoft Corporation, Redmond, Washington, DC, USA) for Windows 11, and a statistical analysis was performed using the software Jamovi project (2022) version 2.3.

Continuous variables were reported as mean and standard deviation.

A repeated measures analysis of variance (ANOVA) was performed to evaluate the effects of different environmental conditions (indoor, outdoor and grass) on gait parameters. The primary aim was to assess whether these environmental conditions had a significant influence on these parameters, as well as to explore potential interactions with demographic and clinical factors such as age, gender, history of previous falls and Tinetti score. These interactions were included within the same model rather than analysed through separate mixed ANOVA or mixed-effects models.

The Greenhouse-Geisser correction was applied to account for violations of the sphericity assumption, as tested by Mauchly’s test. For significant main effects, post hoc pairwise comparisons between environmental conditions (indoor, outdoor, grass) were conducted using a Tukey’s test, which controls for Type I error across multiple comparisons. Additionally, Bonferroni correction was applied to adjust the significance level across multiple ANOVA tests to account for the inflation of Type I error due to repeated measures.

Interaction effects or within-subject effects, including two-way and three-way interactions (e.g., environment ✻ age, environment ✻ gender ✻ previous fall), were also assessed to examine whether the impact of the environment varied based on demographic or clinical characteristics. Conversely, between-subject effects were analysed to determine the influence of fixed factors, including gender and history of previous falls, on gait parameters across all environmental conditions, providing insights into individual and group-level differences. Age and Tinetti score were included as continuous covariates in the ANOVA model rather than categorical factors. Environment was the only within-subject variable, as all participants walked in all three conditions. Age, gender, previous falls and Tinetti score were classified as between-subject factors, since each participant belonged to only one level of these variables. This distinction ensures an appropriate statistical framework for examining both environmental effects and individual differences in gait parameters. Effect sizes were reported as partial eta squared (η^2^p) to quantify the strength of the observed effects. Statistical significance was set at *p* < 0.05.

See Supplementary Materials for complete statistical analysis.

## 3. Results

### 3.1. Population

A total of 99 participants were included in the study, comprising 39 males and 60 females. The characteristics of the participants are presented in Table 1. The mean age of the participants was 55.0 ± 22.7 years. Forty participants reported a history of previous falls. The mean value reported by the Tinetti scale was 20.1 ± 8.9.

### 3.2. Effect of the Environment on Spatio-Temporal Gait Parameters

**Figure 2:**
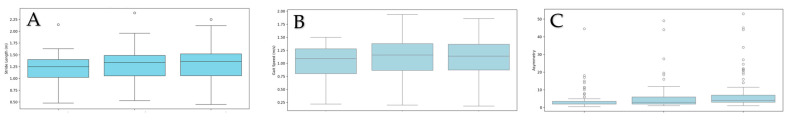
Comparison of spatio-temporal gait parameters in different environments: the figures show an example of how environments affected stride length (**A**), gait speed (**B**) and asymmetry (**C**). In all three graphs, the first boxplot shows values recorded indoors on a flat and clean surface; the second shows those recorded outdoors on a concrete sidewalk; the third presents the values recorded outdoors on grass.

Statistical analysis demonstrated the significant effects of the environment on spatio-temporal gait parameters. Although the main effect of the environment on cadence was not statistically significant (*p* = 0.066) (Table 2), post hoc comparisons using a Tukey’s test revealed significantly lower cadences indoors compared to outdoor environments (*p* < 0.001) (Table 3). Environment significantly influenced stride length (*p* < 0.001) (Table 2, Figure 2A), with shorter strides being found indoors compared to both outdoors (*p* < 0.001) and grass (*p* = 0.007). No significant difference was observed between outdoor and grass environments (*p* = 0.899) (Table 3). Gait speed followed a similar pattern, with significant environmental effects (*p* < 0.001) (Table 2, Figure 2B). Speed was lower in indoor environments than in both outdoors and grass settings (both *p* < 0.001), whereas no significant difference emerged between outdoor and grass environments (*p* = 0.935) (Table 3). Gait asymmetry also varied significantly between environments (*p* < 0.001) (Table 2, Figure 2C). Additionally, although an initial difference in asymmetry between indoor and outdoor environments was observed (uncorrected *p* = 0.027), this effect was no longer significant after applying a Bonferroni correction (*p* = 0.081), whereas indoors and outdoors significantly differing from grass settings (*p* < 0.05) (Table 3). Environmental effects on other parameters, including stride duration, swing phase, single-support, and double-support, were not statistically significant (*p* > 0.05) (Table 2 and Table 3). A Bonferroni correction was applied to control for multiple ANOVA tests, and the corrected *p*-values are reported in Table 3.

### 3.3. Interactions and Between-Subject Effects on Spatio-Temporal Gait Parameters

While no significant interactions were identified between environment and demographic or clinical factors—such as age, gender, previous falls or Tinetti scale—for most parameters, an exception was noted in the interaction between the environment and Tinetti scores for swing phase duration (*p* = 0.048). Additionally, the environment, gender and previous falls interaction was significant for speed (*p* = 0.045) (Table 4).

Between-subject effects consistently highlighted the critical role of the Tinetti scale across gait parameters. Significant associations were observed for cadence (*p* < 0.001), stride length (*p* < 0.001), speed (*p* < 0.001), stride duration (*p* < 0.001), single-support phase (*p* < 0.001) and double-support phase (*p* < 0.001). Conversely, gender, age and previous fall history did not significantly affect any of the parameters (Table 4).

## 4. Discussion

This study showed significant effects of the environment on spatio-temporal gait parameters, highlighting the critical role of environmental conditions in modulating gait dynamics. This finding is consistent with other studies that have demonstrated that indoor cadence, stride length, speed and asymmetry are reduced when compared to outdoor walking [40,41,42], emphasising the restrictive nature of indoor walking spaces. The faster speeds observed outdoors may be due to individuals feeling freer and less restricted in a wide environment compared to the more formal atmosphere of a hospital. Indeed, it may be easier to establish a speed that achieves the desired intensity in a controlled environment [43]. These findings indicate that indoor assessments may not fully reflect functional capacity in real-world settings. This may have important implications for rehabilitation and fall prevention programmes, particularly in the design of environments that promote a realistic setting in order to collect new clinical endpoints and quantitative outcomes. Some authors suggest that longer walking sessions show fewer differences in gait between different groups with different types of dementia [44]. This may be due to the complexity of real-world environments [45]. While laboratory-based gait analysis might be more effective for comparing different groups, real-world assessments are valuable for tracking individual changes, such as disease progression and response to treatment [46].

The present study reported that there were no significant differences between outdoor (on concrete sidewalk) and grass environments for almost all parameters, suggesting that open and natural settings may provide comparable support for functional gait. Additional differences between outdoor and grass environments (*p* = 0.007) were observed only for asymmetry. Specifically, gait asymmetry was most pronounced in grass environments and least evident indoors. Asymmetry is a key variable in assessing gait stability and rhythmicity in relation to fall risk [47]. However, the difference in asymmetry between indoor and outdoor environments did not remain statistically significant after the Bonferroni correction, suggesting that this effect may be less robust than initially observed. It is important to consider that outdoor environments provide different sensory experiences (visual, auditory and tactile) than indoors, which may influence gait control and contribute to increased fall risk and fear of falling in older adults [48,49,50]. The pronounced asymmetry in grass environments may reflect the greater motor demands required to adapt to uneven surfaces, whereas the reduced variability indoors could result in more stable but less adaptable gait patterns. These findings suggest that gait asymmetry may still offer valuable insights into adaptability across different walking contexts, though further investigation is needed to confirm its clinical relevance.

Although the environment influenced several spatio-temporal parameters, no significant effects were observed for the stride duration, swing phase, single-support, or double-support phases. The results suggest that certain specific parameters of gait may be more resilient to variations in the environment. This suggests that these aspects of walking mechanics could be influenced more by inherent biomechanical constraints rather than external factors. Understanding this resistance can provide valuable insights into the stability of gait patterns and highlight the importance of intrinsic physiological factors in maintaining consistent movement regardless of changes in surroundings. However, the interaction between the environment and Tinetti scores for swing phase duration highlights the importance of individual balance abilities in regard to modulating specific gait parameters in response to environmental changes. This aligns with the broader finding that Tinetti scores significantly influenced all gait parameters analysed, reinforcing the role of balance capacity as a critical determinant of gait performance. Our study suggests that gait and balance abilities, evaluated using Tinetti scores, play a more significant role in influencing gait parameters than demographic factors such as age, gender or history of falls. This finding is clinically significant, highlighting the crucial role of the Tinetti scale in identifying people with altered gait parameters, such as cadence (*p* < 0.001), stride length (*p* < 0.001), speed (*p* < 0.001), stride duration (*p* < 0.001), single-support phase (*p* < 0.001) and double-support phase (*p* < 0.001), recorded with a wearable sensor.

This study has some limitations. A significant limitation is the inability to create neurocognitive and psychological profiles of individuals; in fact, spatio-temporal parameters are influenced by cognitive function, psychological factors and emotional well-being [51,52]. For these reasons, further studies which include different levels of cognitive and psychological impairments in their methods are still needed to better investigate changes in gait parameters on various environments. In addition, voluntary selection may introduce bias, as participants may not be representative of the general population. The lack of significant interactions between environmental and clinical factors may be due to insufficient statistical power. Moreover, the use of a repeated measures ANOVA rather than a mixed-effects model is another limitation. While a mixed-effects approach could provide additional insights, our dataset structure (limited repeated measures per subject) may not fully benefit from this method. The study lacks comparisons between different indoor surfaces, such as carpeted and tiled areas. It would also benefit from longitudinal data on gait changes over time, including comparison experiments with other gait assessment tools like the 6-Min Walk Test. The study may be affected by selection bias and does not consider factors like motor disabilities or environmental influences, such as weather and lighting, which can significantly impact walking patterns in real-world situations. Future research should include larger, diverse samples and controls for these variables. In our study, we did not collect specific pain intensity data, as this information served as an inclusion criterion for the recruitment of patients from the podiatry clinic. Future studies should also consider how unstable terrain, like grass, may negatively affect pain and significantly influence gait, in addition to the general effects observed. While this study improves our understanding of how terrain affects people with foot pain, it was conducted in a controlled environment focusing on short-distance straight-line walking. This limits its applicability to real-world situations. Furthermore, the challenges of assessing outdoor walking in clinical settings pose a significant barrier to implementing these findings in practice.

Future research should integrate stability-specific metrics, such as Harmonic Ratio and Margin of Stability, to provide a more comprehensive assessment of gait adaptations and fall risk across different terrains. Additionally, combining wearable sensors with vision-based gait analysis systems (e.g., OpenPose, Microsoft Kinect) could enhance accuracy validation by providing complementary spatial data, including joint angles and posture. A deeper investigation into gait asymmetry origins, considering limb coordination and joint loading, could further clarify biomechanical adaptations to varying environments. Time-series analyses of asymmetry during walking tests and machine learning models (e.g., LSTM) may offer predictive insights into real-time gait adjustments. Furthermore, future studies should explore the generalizability of IMU-based assessments by comparing different sensor brands and evaluating the impact of sensor placement (e.g., lower back vs. shank) on gait asymmetry accuracy.

Despite the limitations, to our knowledge, this is the first study that has investigated the differences in the spatio-temporal parameters during walking between the indoor and two different real-world outdoor environments in such a large sample. The observed environmental effects highlight the need for context-specific gait assessments, as performance in controlled indoor settings may not fully capture a participant’s functional capacity in real-world conditions.

This study provides new insights into sensor-based gait analysis, which is useful for monitoring gait dysfunctions over time and in various clinical fields, where the quantitative assessment of outcomes is crucial for achieving therapy goals.

## 5. Conclusions

This study reveals that gait parameters such as gait speed, stride length and asymmetry vary significantly between indoor and outdoor environments. A wearable gait analysis performed in clinical settings may underestimate spatio-temporal parameters, so clinicians and researchers should be cautious in generalising findings to everyday life.

## Figures and Tables

**Table 1:** Participant’s characteristics.

	Participant (n = 99)
Age	55.0 ± 22.7
Gender	39 M/60 F
Previous Fall	40
Tinetti	20.1 ± 8.9

Abbreviations: M male; F female.

**Table 2:** Spatio-temporal gait parameters according to environment.

	Cadence	Stride Length	Gait Speed	Stride Duration	Asymmetry	Swing Phase	Single-Support	Double-Support
Indoor	99.9 ± 15.8	1.20 ± 0.29	1.02 ± 0.31	1.24 ± 0.25	3.76 ± 5.29	0.52 ± 1.32	0.38 ± 0.05	0.25 ± 0.1
Outdoor	103 ± 17.3	1.27 ± 0.33	1.11 ± 0.37	1.22 ± 0.32	5.38 ± 7.36	0.38 ± 0.05	0.38 ± 0.05	0.24 ± 0.1
Grass	101 ± 20.6	1.29 ± 0.36	1.11 ± 0.39	1.25 ± 0.36	7.31 ± 9.45	0.38 ± 0.06	0.38 ± 0.06	0.25 ± 0.12
F (df)	3.13 (1.37)	11.35 (1.36)	25.45 (1.72)	1.25 (1.72)	16.71 (1.71)	1.03 (1.00)	2.35 (1.82)	2.46 (1.73)
*p*-value	0.066	**<0.001** *	**<0.001** *	0.285	**<0.001** *	0.313	0.103	0.096
η^2^p	0.033	0.109	0.215	0.013	0.152	0.011	0.025	0.026

η^2^p: effect sizes, df: degree of freedom; F: F-statistics. * Indicates reaching statistical significance (*p* < 0.05).

**Table 3:** Post Hoc comparisons for different environment settings.

	Cadence	Stride Length	Gait Speed	Stride Duration	Asymmetry	Swing Phase	Single-Support	Double-Support
indoor-outdoor	**<0.001** (<0.001) *	**<0.001** (<0.001) *	**<0.001** (<0.001) *	0.402 (1.000)	0.027 (<0.081)	0.788 (1.000)	0.334 (1.000)	0.407 (1.000)
indoor-grass	0.805 (1.000)	**0.007** (0.021) *	**<0.001** (<0.001) *	0.919 (1.000)	**<0.001** (<0.001) *	0.775 (1.000)	0.968 (1.000)	0.84 (1.000)
outdoor-grass	0.406 (1.000)	0.899 (1.000)	0.935 (1.000)	0.853 (1.000)	**0.007** (<0.021) *	0.606 (1.000)	0.157 (0.471)	0.084 (0.252)

* Indicates reaching statistical significance (*p* < 0.05). A Tukey’s test was used for post hoc pairwise comparisons between environmental conditions (indoor, outdoor, grass). A Bonferroni correction was applied to adjust for multiple ANOVA tests, and corrected *p*-values are reported in round brackets.

**Table 4:** Significance of within- and between-subject effects on spatio-temporal gait parameters.

		Cadence	Stride Length	Gait Speed	Stride Duration	Asymmetry	Swing Phase	Single-Support	Double-Support
**Within subjects effects**	Environment	0.066 (0.033)	<0.001 * (0.109)	<0.001 * (0.215)	0.285 (0.013)	<0.001 * (0.152)	0.313 (0.011)	0.103 (0.025)	0.096 (0.026)
	Environment ✻ age	0.683 (0.003)	0.494 (0.006)	0.562 (0.006)	0.202 (0.017)	0.284 (0.013)	0.179 (0.019)	0.544 (0.006)	0.414 (0.009)
	Environment ✻ Tinetti	0.801 (0.001)	0.311 (0.012)	0.514 (0.007)	0.872 (0.001)	0.153 (0.020)	0.048 * (0.041)	0.219 (0.016)	0.164 (0.020)
	Environment ✻ gender	0.187 (0.018)	0.626 (0.004)	0.606 (0.005)	0.116 (0.024)	0.707 (0.003)	0.489 (0.005)	0.221 (0.016)	0.069 (0.030)
	Environment ✻ previous fall	0.417 (0.008)	0.498 (0.006)	0.582 (0.005)	0.361 (0.011)	0.804 (0.002)	0.924 (0.000)	0.823 (0.002)	0.619 (0.005)
	Environment ✻ gender ✻ previous fall	0.059 (0.034)	0.231 (0.016)	0.045 * (0.035)	0.793 (0.002)	0.333 (0.012)	0.221 (0.016)	0.271 (0.014)	0.097 (0.026)
**Between subjects effects**	Gender	0.439 (0.006)	0.073 (0.034)	0.683 (0.002)	0.899 (0.000)	0.749 (0.001)	0.516 (0.005)	0.996 (0.000)	0.948 (0.000)
	Previous fall	0.773 (0.001)	0.551 (0.004)	0.559 (0.004)	0.708 (0.002)	0.535 (0.004)	0.91 (0.000)	0.505 (0.005)	0.748 (0.001)
	Gender ✻ previous fall	0.092 (0.030)	0.643 (0.002)	0.191 (0.018)	0.144 (0.023)	0.186 (0.019)	0.261 (0.014)	0.79 (0.001)	0.531 (0.004)
	Age	0.285 (0.012)	0.684 (0.002)	0.325 (0.010)	0.339 (0.010)	0.559 (0.004)	0.186 (0.019)	0.657 (0.002)	0.432 (0.007)
	Tinetti	<0.001 * (0.114)	<0.001 * (0.208)	<0.001 * (0.0261)	<0.001 * (0.0124)	0.155 (0.022)	0.111 (0.027)	<0.001 * (0.224)	<0.001 * (0.227)

Note: Effect sizes (η^2^p) are reported in round brackets. * Indicates reaching statistical significance (*p* < 0.05).

## Data Availability

The data presented in this study are available on request from the corresponding author; however, restrictions apply to the availability of these data, which were used under licence for this study, and they are thus not publicly available.

## Supplementary Materials

The following supporting information can be downloaded at https://www.mdpi.com/article/10.3390/s25051314/s1. Comprehensive statistical data analysis.
