# Supplementary material for: Gait Spatio-Temporal Parameters Vary Significantly Between Indoor, Outdoor and Different Surfaces
Source: Sensors (Basel). 2025 Feb 21;25(5):1314. doi: 10.3390/s25051314 (PMC11902731; doi:10.3390/s25051314)
Supplement: Supplementary file 1 [file sensors-25-01314-s001.zip › sensors-3431379-supplementary.pdf]

Cadence

Repeated Measures ANOVA

Within Subjects Effects

	Sphericity Correction	Sum of Squares	df	Mean Square	F	p	η^2_p
Enviroment	Greenhouse-Geisser	406.5	1.37	297.4	3.128	0.066	0.033
Enviroment ✻ Age	Greenhouse-Geisser	34.2	1.37	25.0	0.263	0.683	0.003
Enviroment ✻ Tinetti	Greenhouse-Geisser	16.3	1.37	11.9	0.126	0.801	0.001
Enviroment ✻ Gender	Greenhouse-Geisser	227.6	1.37	166.5	1.752	0.187	0.018
Enviroment ✻ Previous fall	Greenhouse-Geisser	101.1	1.37	73.9	0.778	0.417	0.008
Enviroment ✻ Gender ✻ Previous fall	Greenhouse-Geisser	424.8	1.37	310.8	3.270	0.059	0.034
Residual	Greenhouse-Geisser	12082.9	127.12	95.1			

Note. Type 2 Sums of Squares

Between Subjects Effects

	Sum of Squares	df	Mean Square	F	p	η^2_p
Gender	379.7	1	379.7	0.6044	0.439	0.006
Previous fall	52.6	1	52.6	0.0836	0.773	0.001
Gender ✻ Previous fall	1825.1	1	1825.1	2.9050	0.092	0.030
Age	725.3	1	725.3	1.1544	0.285	0.012
Tinetti	7496.9	1	7496.9	11.9330	< .001	0.114
Residual	58427.2	93	628.2			

Note. Type 2 Sums of Squares

Assumptions

Tests of Sphericity

	Mauchly's W	p	Greenhouse-Geisser ε	Huynh-Feldt ε
Enviroment	0.537	< .001	0.683	0.690

Post Hoc Tests

Post Hoc Comparisons - Enviroment

Comparison							
**Enviroment**		**Enviroment**	**Mean Difference**	**SE**	**df**	**t**	**p_{tukey}**
Indoor	-	Outdoor	-2.694	0.720	93.0	-3.742	< .001
	-	Grass	-0.944	1.501	93.0	-0.629	0.805
Outdoor	-	Grass	1.750	1.360	93.0	1.287	0.406

Stride length

Repeated Measures ANOVA

Within Subjects Effects

	Sphericity Correction	Sum of Squares	df	Mean Square	F	p	η^2_p
Enviroment	Greenhouse-Geisser	0.3835	1.36	0.28257	11.356	< .001	0.109
Enviroment ✻ Age	Greenhouse-Geisser	0.0198	1.36	0.01461	0.587	0.494	0.006
Enviroment ✻ Tinetti	Greenhouse-Geisser	0.0378	1.36	0.02785	1.119	0.311	0.012
Enviroment ✻ Gender	Greenhouse-Geisser	0.0116	1.36	0.00852	0.343	0.626	0.004
Enviroment ✻ Previous fall	Greenhouse-Geisser	0.0195	1.36	0.01439	0.578	0.498	0.006
Enviroment ✻ Gender ✻ Previous fall	Greenhouse-Geisser	0.0501	1.36	0.03691	1.483	0.231	0.016
Residual	Greenhouse-Geisser	3.1405	126.20	0.02488			

Note. Type 2 Sums of Squares

Between Subjects Effects

	Sum of Squares	df	Mean Square	F	p	η^2_p
Gender	0.4450	1	0.4450	3.283	0.073	0.034
Previous fall	0.0486	1	0.0486	0.358	0.551	0.004
Gender ✻ Previous fall	0.0293	1	0.0293	0.216	0.643	0.002
Age	0.0225	1	0.0225	0.166	0.684	0.002
Tinetti	3.3070	1	3.3070	24.399	< .001	0.208
Residual	12.6053	93	0.1355			

Note. Type 2 Sums of Squares

Assumptions

Tests of Sphericity

	Mauchly's W	p	Greenhouse-Geisser ε	Huynh-Feldt ε
Enviroment	0.526	< .001	0.679	0.685

Post Hoc Tests

Post Hoc Comparisons - Enviroment

Comparison							
**Enviroment**		**Enviroment**	**Mean Difference**	**SE**	**df**	**t**	p_{tukey}
Indoor	-	Outdoor	-0.06537	0.0113	93.0	-5.765	< .001
	-	Grass	-0.07516	0.0240	93.0	-3.132	0.007
Outdoor	-	Grass	-0.00979	0.0223	93.0	-0.439	0.899

Gait speed

Repeated Measures ANOVA

Within Subjects Effects

	Sphericity Correction	Sum of Squares	df	Mean Square	F	p	η^2_p
Enviroment	Greenhouse-Geisser	0.53792	1.72	0.31348	25.454	< .001	0.215
Enviroment ✻ Age	Greenhouse-Geisser	0.01120	1.72	0.00653	0.530	0.562	0.006
Enviroment ✻ Tinetti	Greenhouse-Geisser	0.01317	1.72	0.00768	0.623	0.514	0.007
Enviroment ✻ Gender	Greenhouse-Geisser	0.00958	1.72	0.00558	0.453	0.606	0.005
Enviroment ✻ Previous fall	Greenhouse-Geisser	0.01047	1.72	0.00610	0.495	0.582	0.005
Enviroment ✻ Gender ✻ Previous fall	Greenhouse-Geisser	0.07060	1.72	0.04114	3.341	0.045	0.035
Residual	Greenhouse-Geisser	1.96534	159.58	0.01232			

Note. Type 2 Sums of Squares

Between Subjects Effects

	Sum of Squares	df	Mean Square	F	p	η^2_p
Gender	0.0267	1	0.0267	0.168	0.683	0.002
Previous fall	0.0547	1	0.0547	0.345	0.559	0.004
Gender ✻ Previous fall	0.2747	1	0.2747	1.731	0.191	0.018
Age	0.1554	1	0.1554	0.979	0.325	0.010
Tinetti	5.2246	1	5.2246	32.925	< .001	0.261
Residual	14.7573	93	0.1587			

Note. Type 2 Sums of Squares

Assumptions

Tests of Sphericity

	Mauchly's W	p	Greenhouse-Geisser ε	Huynh-Feldt ε
Enviroment	0.834	< .001	0.858	0.873

Post Hoc Tests

Post Hoc Comparisons - Enviroment

Comparison							
**Enviroment**		**Enviroment**	**Mean Difference**	**SE**	**df**	**t**	**p_{tukey}**
Indoor	-	Outdoor	-0.08627	0.0135	93.0	-6.393	< .001
	-	Grass	-0.09144	0.0187	93.0	-4.887	< .001
Outdoor	-	Grass	-0.00518	0.0148	93.0	-0.350	0.935

Stride duration

Repeated Measures ANOVA

Within Subjects Effects

	Sphericity Correction	Sum of Squares	df	Mean Square	F	p	η^2_p
Enviroment	Greenhouse-Geisser	0.04988	1.72	0.02907	1.253	0.285	0.013
Enviroment ✻ Age	Greenhouse-Geisser	0.06506	1.72	0.03792	1.635	0.202	0.017
Enviroment ✻ Tinetti	Greenhouse-Geisser	0.00418	1.72	0.00244	0.105	0.872	0.001
Enviroment ✻ Gender	Greenhouse-Geisser	0.08982	1.72	0.05235	2.257	0.116	0.024
Enviroment ✻ Previous fall	Greenhouse-Geisser	0.03964	1.72	0.02310	0.996	0.361	0.011
Enviroment ✻ Gender ✻ Previous fall	Greenhouse-Geisser	0.00761	1.72	0.00444	0.191	0.793	0.002
Residual	Greenhouse-Geisser	3.70176	159.56	0.02320			

Note. Type 2 Sums of Squares

Between Subjects Effects

	Sum of Squares	df	Mean Square	F	p	η^2_p
Gender	0.00320	1	0.00320	0.0163	0.899	0.000
Previous fall	0.02769	1	0.02769	0.1413	0.708	0.002
Gender ✻ Previous fall	0.42433	1	0.42433	2.1661	0.144	0.023
Age	0.18108	1	0.18108	0.9243	0.339	0.010
Tinetti	2.58864	1	2.58864	13.2140	< .001	0.124
Residual	18.21878	93	0.19590			

Note. Type 2 Sums of Squares

Assumptions

Tests of Sphericity

	Mauchly's W	p	Greenhouse-Geisser ε	Huynh-Feldt ε
Enviroment	0.834	< .001	0.858	0.872

Post Hoc Tests

Post Hoc Comparisons - Enviroment

Comparison							
**Enviroment**		**Enviroment**	**Mean Difference**	**SE**	**df**	**t**	**p_{tukey}**
Indoor	-	Outdoor	0.02218	0.0171	93.0	1.294	0.402
	-	Grass	0.00875	0.0223	93.0	0.392	0.919
Outdoor	-	Grass	-0.01343	0.0250	93.0	-0.538	0.853

Asymmetry

Repeated Measures ANOVA

Within Subjects Effects

	Sphericity Correction	Sum of Squares	df	Mean Square	F	p	η^2_p
Enviroment	Greenhouse-Geisser	623.40	1.71	364.88	16.713	< .001	0.152
Enviroment ✻ Age	Greenhouse-Geisser	46.89	1.71	27.44	1.257	0.284	0.013
Enviroment ✻ Tinetti	Greenhouse-Geisser	72.54	1.71	42.46	1.945	0.153	0.020
Enviroment ✻ Gender	Greenhouse-Geisser	11.15	1.71	6.53	0.299	0.707	0.003
Enviroment ✻ Previous fall	Greenhouse-Geisser	6.58	1.71	3.85	0.176	0.804	0.002
Enviroment ✻ Gender ✻ Previous fall	Greenhouse-Geisser	40.44	1.71	23.67	1.084	0.333	0.012
Residual	Greenhouse-Geisser	3469.01	158.89	21.83			

Note. Type 2 Sums of Squares

Between Subjects Effects

	Sum of Squares	df	Mean Square	F	p	η^2_p
Gender	13.1	1	13.1	0.103	0.749	0.001
Previous fall	49.2	1	49.2	0.387	0.535	0.004
Gender ✻ Previous fall	225.6	1	225.6	1.773	0.186	0.019
Age	43.7	1	43.7	0.344	0.559	0.004
Tinetti	261.9	1	261.9	2.058	0.155	0.022
Residual	11831.7	93	127.2			

Note. Type 2 Sums of Squares

Assumptions

Tests of Sphericity

	Mauchly's W	p	Greenhouse-Geisser ε	Huynh-Feldt ε
Enviroment	0.829	< .001	0.854	0.869

Post Hoc Tests

Post Hoc Comparisons - Enviroment

Comparison							
**Enviroment**		**Enviroment**	**Mean Difference**	**SE**	**df**	**t**	**p_{tukey}**
Indoor	-	Outdoor	-1.60	0.607	93.0	-2.63	0.027
	-	Grass	-3.40	0.790	93.0	-4.31	< .001
Outdoor	-	Grass	-1.81	0.578	93.0	-3.12	0.007

Swing phase

Repeated Measures ANOVA

Within Subjects Effects

	Sphericity Correction	Sum of Squares	df	Mean Square	F	p	η^2_p
Enviroment	Greenhouse-Geisser	1.2022	1.00	1.2012	1.02975	0.313	0.011
Enviroment ✻ Age	Greenhouse-Geisser	2.1429	1.00	2.1411	1.83556	0.179	0.019
Enviroment ✻ Tinetti	Greenhouse-Geisser	4.6873	1.00	4.6834	4.01503	0.048	0.041
Enviroment ✻ Gender	Greenhouse-Geisser	0.5626	1.00	0.5621	0.48192	0.489	0.005
Enviroment ✻ Previous fall	Greenhouse-Geisser	0.0108	1.00	0.0107	0.00921	0.924	0.000
Enviroment ✻ Gender ✻ Previous fall	Greenhouse-Geisser	1.7736	1.00	1.7721	1.51923	0.221	0.016
Residual	Greenhouse-Geisser	108.5713	93.08	1.1665			

Note. Type 2 Sums of Squares

Between Subjects Effects

	Sum of Squares	df	Mean Square	F	p	η^2_p
Gender	0.23548	1	0.23548	0.4261	0.516	0.005
Previous fall	0.00717	1	0.00717	0.0130	0.910	0.000
Gender ✻ Previous fall	0.70614	1	0.70614	1.2776	0.261	0.014
Age	0.98175	1	0.98175	1.7763	0.186	0.019
Tinetti	1.43361	1	1.43361	2.5939	0.111	0.027
Residual	51.40029	93	0.55269			

Note. Type 2 Sums of Squares

Assumptions

Tests of Sphericity

	Mauchly's W	p	Greenhouse-Geisser ε	Huynh-Feldt ε
Enviroment	0.00168	< .001	0.500	0.500

Post Hoc Tests

Post Hoc Comparisons - Enviroment

Comparison							
**Enviroment**		**Enviroment**	**Mean Difference**	**SE**	**df**	**t**	**p_{tukey}**
Indoor	-	Outdoor	0.09485	0.14412	93.0	0.658	0.788
	-	Grass	0.09811	0.14398	93.0	0.681	0.775
Outdoor	-	Grass	0.00326	0.00341	93.0	0.956	0.606

Single support

Repeated Measures ANOVA

Within Subjects Effects

	Sphericity Correction	Sum of Squares	df	Mean Square	F	p	η^2_p
Enviroment	Greenhouse-Geisser	0.00277	1.82	0.00152	2.356	0.103	0.025
Enviroment ✻ Age	Greenhouse-Geisser	6.86e-4	1.82	3.77e-4	0.583	0.544	0.006
Enviroment ✻ Tinetti	Greenhouse-Geisser	0.00181	1.82	9.94e-4	1.540	0.219	0.016
Enviroment ✻ Gender	Greenhouse-Geisser	0.00180	1.82	9.87e-4	1.528	0.221	0.016
Enviroment ✻ Previous fall	Greenhouse-Geisser	2.01e-4	1.82	1.10e-4	0.171	0.823	0.002
Enviroment ✻ Gender ✻ Previous fall	Greenhouse-Geisser	0.00154	1.82	8.46e-4	1.310	0.271	0.014
Residual	Greenhouse-Geisser	0.10941	169.44	6.46e-4			

Note. Type 2 Sums of Squares

Between Subjects Effects

	Sum of Squares	df	Mean Square	F	p	η^2_p
Gender	9.14e-8	1	9.14e-8	2.02e-5	0.996	0.000
Previous fall	0.00202	1	0.00202	0.4471	0.505	0.005
Gender ✻ Previous fall	3.23e-4	1	3.23e-4	0.0714	0.790	0.001
Age	8.97e-4	1	8.97e-4	0.1983	0.657	0.002
Tinetti	0.12132	1	0.12132	26.8057	< .001	0.224
Residual	0.42090	93	0.00453			

Note. Type 2 Sums of Squares

Assumptions

Tests of Sphericity

	Mauchly's W	p	Greenhouse-Geisser ε	Huynh-Feldt ε
Enviroment	0.902	0.009	0.911	0.928

Post Hoc Tests

Post Hoc Comparisons - Enviroment

Comparison							
**Enviroment**		**Enviroment**	**Mean Difference**	**SE**	**df**	**t**	**p_{tukey}**
Indoor	-	Outdoor	-0.00505	0.00360	93.0	-1.402	0.344
	-	Grass	0.00104	0.00426	93.0	0.245	0.968
Outdoor	-	Grass	0.00609	0.00328	93.0	1.859	0.157

Double support

Repeated Measures ANOVA

Within Subjects Effects

	Sphericity Correction	Sum of Squares	df	Mean Square	F	p	η^2_p
Enviroment	Greenhouse-Geisser	0.00934	1.73	0.00539	2.468	0.096	0.026
Enviroment ✻ Age	Greenhouse-Geisser	0.00323	1.73	0.00186	0.852	0.414	0.009
Enviroment ✻ Tinetti	Greenhouse-Geisser	0.00705	1.73	0.00407	1.863	0.164	0.020
Enviroment ✻ Gender	Greenhouse-Geisser	0.01074	1.73	0.00620	2.838	0.069	0.030
Enviroment ✻ Previous fall	Greenhouse-Geisser	0.00165	1.73	9.50e-4	0.435	0.619	0.005
Enviroment ✻ Gender ✻ Previous fall	Greenhouse-Geisser	0.00928	1.73	0.00536	2.453	0.097	0.026
Residual	Greenhouse-Geisser	0.35198	161.22	0.00218			

Note. Type 2 Sums of Squares

Between Subjects Effects

	Sum of Squares	df	Mean Square	F	p	η^2_p
Gender	7.33e-5	1	7.33e-5	0.00425	0.948	0.000
Previous fall	0.00180	1	0.00180	0.10416	0.748	0.001
Gender ✻ Previous fall	0.00682	1	0.00682	0.39503	0.531	0.004
Age	0.01076	1	0.01076	0.62343	0.432	0.007
Tinetti	0.47171	1	0.47171	27.32303	< .001	0.227
Residual	1.60556	93	0.01726			

Note. Type 2 Sums of Squares

Assumptions

Tests of Sphericity

	Mauchly's W	p	Greenhouse-Geisser ε	Huynh-Feldt ε
Enviroment	0.846	< .001	0.867	0.882

Post Hoc Tests

Post Hoc Comparisons - Enviroment

Comparison							
**Enviroment**		**Enviroment**	**Mean Difference**	**SE**	**df**	**t**	**p_{tukey}**
Indoor	-	Outdoor	0.00803	0.00624	93.0	1.285	0.407
	-	Grass	-0.00444	0.00788	93.0	-0.563	0.840
Outdoor	-	Grass	-0.01246	0.00578	93.0	-2.155	0.084
